# Appearance of E1: A226V mutant Chikungunya virus in Coastal Karnataka, India during 2008 outbreak

**DOI:** 10.1186/1743-422X-6-172

**Published:** 2009-10-27

**Authors:** SR Santhosh, Paban Kumar Dash, Manmohan Parida, Mohasin Khan, Putcha VL Rao

**Affiliations:** 1 Division of Virology, Defence R & D Establishment (DRDE), Jhansi Road, Gwalior, MP, PIN - 474 002, India

## Abstract

Chikungunya has resurged in the form of unprecedented explosive epidemic in 2006 after a long gap in India affecting 1.39 million of persons. The disease continued for the next two consecutive years affecting 59,535 and 64,548 persons during 2007 and 2008 respectively. The 2008 outbreak being the second largest among these three years the information regarding the etiology and the mutations involved are useful for further control measures. Among the 2008 outbreaks the Coastal Karnataka accounts for the 46,510 persons. An in-depth investigation of Chikungunya epidemic of Coastal Karnataka, India, 2008 by serology, virus isolation, RT-PCR and genome sequencing revealed the presence and continued circulation of A226V mutant Chikungunya virus. The appearance of this mutant virus was found to be associated with higher prevalence of vector *Aedes albopictus *and the geographical proximity of coastal Karnataka with the adjoining Kerala state. This is the first report regarding the appearance of this mutation in Karnataka state of India. The present study identified the presence and association of A226V mutant virus with Chikungunya outbreak in India during 2008.

## Findings

Chikungunya fever is an acute arthropod borne viral illness reported from many parts of Africa and south east Asia. The causative agent is Chikungunya virus (CHIKV), a member of the genus *Alphavirus *of the family *Togaviridae *and is primarily transmitted by the *Aedes aegypti *mosquito [1-3]. CHIKV illness in humans is often characterized by sudden onset of fever, headache, fatigue, nausea, vomiting, rash, myalgia and severe arthralgia. The arthralgia may persist in a small proportion of cases even for months. These clinical symptoms mimic with that of dengue fever and therefore, many cases of Chikungunya are misdiagnosed as dengue infections [4-6]. At present, there is no vaccine or antiviral therapy available against Chikungunya infection.

An outbreak of Chikungunya virus infection occurred during 2006 in 15 states or union territories in India affecting more than 1.39 million of persons [7-10]. The epidemic started from December 2005 and since then continued for the next three consecutive years. Among the various outbreaks in different states, the 2007 outbreak of Kerala was unique in the sense that it affected more than 25,000 persons with higher epidemic potential and reported mortalities [11]. The investigation of full genome sequence of 2007 Kerala isolates and its comparison with 2006 Indian isolates revealed the presence of A226V mutation in E1 gene of the virus and was found to be associated with evolutionary success due to adaptation in the *Aedes albopictus *mosquito vector with progression of epidemic from 2006 to 2007 [11,12].

In 2008, an outbreak of fever with severe arthralgia occurred in costal Karnataka, in India adjoining the state of Kerala, affecting 46,510 persons [10]. The affected areas include Puttur, Mangalore, Sulya and other parts of the Dakshina Kannada district. The outbreak seems similar to Kerala 2007 outbreak with respect to higher epidemic potential and reported mortalities. The geographical proximity of coastal Karnataka to Kerala coupled with the higher prevalence of *Aedes albopictus *created an apprehension regarding involvement of similar etiology. To rule out any confusion, an in depth virological, serological and molecular investigation was carried out to identify the etiology of this unprecedented outbreak, which is considered to be the second highest in terms of number of persons affected since its resurgence in 2006.

A total of 100 blood samples from patients suspected of having Chikungunya fever were brought from District Surveillance Unit, Mangalore which were collected from Mangalore, Puttur, Sulya and other parts of the Dakshina Kannada district for this study. Two sets of blood samples were collected with and without anticoagulant for virus isolation and serology respectively. All these samples were investigated for the presence of Chikungunya specific RNA by RT-PCR and for the presence of CHIKV specific IgM antibodies by using recombinant E1 and E2 protein based IgM ELISA.

The presence of CHIKV specific RNA in clinical samples was detected using the Access quick one-step RT-PCR kit (Promega, USA) employing a primers pair targeting the E1 gene (CHIK13: TTACATCACGTGCGAATAC genome position 10128-10146 and CHIK14: CTTTGCTCTCAGGCGTGCGACTTT genome position 10604-10627); designed from the nucleotide sequence of the reference S27 strain, GenBank Acc No. AF490259[8,11]. The 8 representative RT-PCR positive samples were again amplified through RT-PCR using a different set of primer targeting E1 gene (E1-10145F:ACAAAACCGTCATCCCGTCTC genome position 10145-10165, E1-11158R: TGACTATGTGGTCCTTCGGAGG genome position 11137-11158, E1-11011F:CGGGAAGCTGAGATAGAAGTTGAA Genome position 11011-11034, 3'NTR-11669R: TTGATTTTTATTAGTTTTATGTTT genome position 11645-11669) for sequencing and were subjected to double stranded sequencing employing Big dye terminator cycle sequencing ready reaction kit with ABI 310 sequencer (Applied Biosystems, USA). The nucleotide sequences were aligned edited and analysed using Seqscape V.3 software (Applied Biosystem, USA). CLUSTALW version 1.83 [13] was used to perform multiple nucleotide and amino acid sequence allignment of E1 gene (1044 nt). A The phylogenetic analysis was performed based on partial E1 gene (837 nt) sequences of CHIK viruses using MEGA version 3.1 [14].

For the construction of phylogenetic trees, the neighbour-joining algorithm and the Kimura two-parameter distance modelwere utilized. The reliability of the analysiswas evaluated by a bootstrap test with 10,000 replications

The isolation of virus was also attempted in C_6/36 _cells from selected RT-PCR positive samples following the virus adsorption technique [15]. The serological analysis was carried out using an in-house developed recombinant E1 & E2 protein based indirect format IgM ELISA.

The serological analysis of the samples indicated overall 28% seropositivity for IgM antibodies. A total of 40 (40%) serum samples were found positive for the presence of CHIKV specific RNA, through demonstration of CHIKV specific 500 bp amplicon by RT-PCR. A representative of 20 RT-PCR positive samples were subjected to virus isolation in C_6/36 _cells, which yielded 8 CHIKV isolates. The isolation of the virus was further confirmed by RT-PCR and nucleotide sequencing.

**Figure 1:**
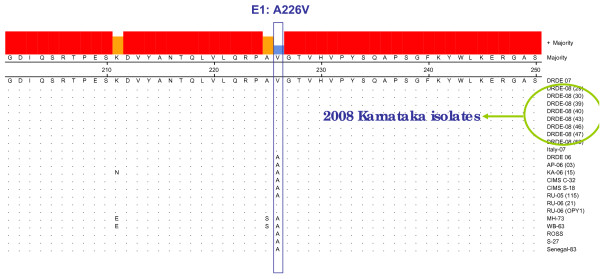
**Showing portion of alignment of amino acid sequences of the E1 gene of CHIKV isolates (amino acid positions from E1: 201-250 are shown)**. The position of the A226V mutation is indicated by an vertical column. Sequences are identified by the name as given in the table 1.

**Figure 2:**
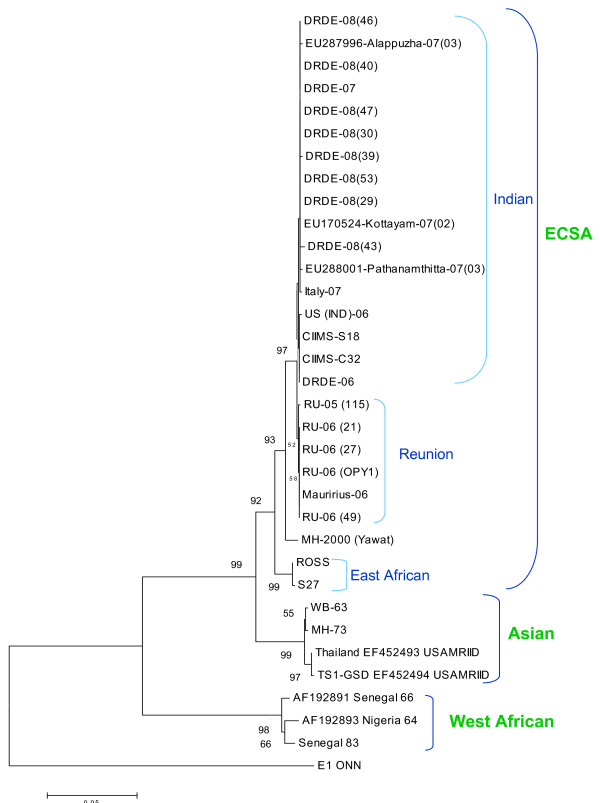
**Phylogenetic tree among Chikungunya viruses generated by neighbourjoining method based on the nucleotide sequence of Partial E1 gene of 35 isolates**. Numbers at nodes indicate bootstrap support (%). The details of the isolates in the figure are described in table 1.

Nucleotide sequencing of the partial E1 gene of 8 representative CHIKV strains was determined and were compared with 27 other globally diverse CHIKV isolates including Chikungunya isolates from 2006, 2007 outbreaks of India and Reunion islands (Table 1). The BLAST search revealed > 99% identity with CHIKV isolates from 2006-07, French Reunion isolates. All the Eight representative isolates from this outbreak revealed A226V shift in the E1 gene as observed in the 2007 Kerala isolates (Fig. 1). The presence of this A226V shift in 2008, coastal Karnataka isolates as well as the geographical proximity with adjoining karala state where this mutation was reported and the results of phylogenetic analysis suggest that the current outbreak might have spread from the Kerala. As E1 gene sequences were available for additional isolates and also because of its importance in phylogenetic analysis, a Neighbour-joining phylogenetic was constructed (Fig. 2). It revealed that all the DRDE-08 isolates from this epidemic grouped along with the DRDE-07, and other 2007 Kerala isolates within the Indian Cluster of ECSA genotype, whereas all the Reunion isolates form a RU cluster with in the ECSA genotype as reported earlier [11].

**Table 1:** Description of CHIKV Isolates from diverse geographical origin used in this study

**Sl. No**	**Virus ID**	**Year of Collection**	**Place**	**Genotype**	**GenBank Acc. No**
1	DRDE 06	2006	AP, India	ECSA	EF210157
2	DRDE 07	2007	Kerala, India	ECSA	EU372006
3	US IND 06	2006	US	ECSA	EF187887
4	RU 06 21	2006	Reunion	ECSA	AM258992
5	RU 06 27	2006	Reunion	ECSA	AM258993
6	RU 06 49	2006	Reunion	ECSA	AM258994
7	RU 05 115	2005	Reunion	ECSA	AM258990
8	RU 05 209	2005	Reunion	ECSA	AM258991
9	Maurititus 06	2006	Maurititus	ECSA	EF187893
10	RU 06 OPY1	2006	Reunion	ECSA	DQ443544
12	MH 2000Yawat	2000	Yawat, MH, India	ECSA	EF027139
13	ROSS	1953	Tanzania	ECSA	AF490259
14	S 27	1953	Tanzania	ECSA	NC_004162
15	MH 73	1973	MH, India	Asian	EF027141
16	WB 63	1963	WB, India	Asian	EF027140
17	Senegal 66	1966	Senegal	West Africa	AF192891
18	Nigeria 64	1964	Nigeria	West Africa	AF192893
19	Senegal 83	1983	Senegal	West Africa	AY726732
20	Italy 07	2007	Italy	ECSA	EU244823
21	DRDE 08 53	2008	Karnataka, India	ECSA	GQ996377
22	DRDE 08 47	2008	Karnataka, India	ECSA	GQ996376
23	DRDE 08 46	2008	Karnataka, India	ECSA	GQ996375
24	DRDE 08 43	2008	Karnataka, India	ECSA	GQ996374
25	DRDE 08 40	2008	Karnataka, India	ECSA	GQ996373
26	DRDE 08 39	2008	Karnataka, India	ECSA	GQ996372
27	DRDE 08 30	2008	Karnataka, India	ECSA	GQ996371
28	DRDE 08 29	2008	Karnataka, India	ECSA	GQ996370
29	CIIMS 06-S18	2006	Nagpur, India	ECSA	GQ996379
30	CIIMS 06-C32	2006	Nagpur, India	ECSA	GQ996378
31	TS1GSDUSARMID	2007*	USA	Asian	EF452494
32	ThailandUSARMID	-	Thailand	Asian	EF452493
33	Alapuzha-07(03)	2007	Kerala	ECSA	EU287996
34	Alapuzha-07(03)	2007	Kerala	ECSA	EU170524
35	Pathanamthitta-07(03)	2007	Kerala	ECSA	EU288001

Since early 2005, a major epidemic of CHIK started in many Indian Ocean island nations; and towards end of 2005, it reemerged in several parts of India [7-9,16]. The resurgence of CHIK epidemic after a gap of 32 years and the subsequent hiatus of explosive epidemic for three consecutive years is a point of major concern. In addition, the implication of A226V mutation with increased severity, non classical symptoms, reported mortality and large epidemic in Kerala during 2007 has warranted the continues monitoring and surveillance of the activity of the mutant virus in this particular region [11]. The investigation of 2008 coastal Karnataka outbreak clearly showed the involvement of A226V mutant in large scale morbidity.

The present study revealed less seropositivity in comparison to RT-PCR which may be attributed to the collection of samples at very early or acute stage of the illness. The demonstration of CHIKV RNA in 40% samples by RT-PCR and detection of IgM antibodies in 28% of sample confirmed the causative agent of this epidemic to be CHIKV. The isolation of CHIKV from clinical samples further confirmed this etiology. The sequence of CHIKV were directly determined from clinical samples without risk of altering the genome by *in vitro *passaging. During this outbreak, patients also reported non classical symptoms including hemorrhage, lymphadenitis, ictures and liver involvement, etc. similar to the cases in Reunion 2005-06 and Kerala India-2007 [11,16]. These types of unusual cases, geographical similarity, proximity and higher prevalence of *Aedes albopictus *leads to the speculation for the involvement of Kerala 2007 strain for the current outbreak. The sequencing of CHIKV strains from the current 2008 outbreak led to identification of A226V shift in these isolates. The E1:A226V mutation was earlier correlated with vector specificity as well as epidemic potential [17]. This shift in the present outbreak may be attributed to the higher epidemic potential and higher prevalence of *Aedes albopictus *vector in Coastal Karnataka in 2008. However, the complete genome sequencing is required to see other mutations other than A226V which are unique for the current 2008 outbreak

The phylogenetic analysis revealed that all isolates from the current outbreak were very closely related to analogous strains from Kerala 2007 outbreak. All these isolates harbor valine at E1-226 position compared to alanine in the 2006 Indian isolates

In summary, to the best of our knowledge, this is the first report regarding the appearance of this mutation in Karnataka state of India. The involvement of A226V mutant virus was attributed to the continued circulation of the 2007 Kerala strain in the current outbreak due its geographical proximity coupled with higher prevalence of *Aedes albopictus *vector, supporting the higher epidemic potential of A226V mutant virus. However, a continuous surveillance is warranted to monitor its spread and track possible evolution of the virus during the epidemic.

## Competing interests

The authors declare that they have no competing interests.

## Authors' contributions

SRS defined the study and carried out the laboratory experiments, interpreted the results and wrote the manuscript. PKD designed the primers, analyzed the data. MMP co-interpreted the results and co-wrote the manuscript. PVLR and MK contributed their ideas to the design of the study and the manuscript. All authors read and approved the final manuscript.
